# Supplementary figures and images for: Chicken Immune Cell Assay to Model Adaptive Immune Responses In Vitro
Source: Animals (Basel). 2021 Dec 19;11(12):3600. doi: 10.3390/ani11123600 (PMC8697874; doi:10.3390/ani11123600)

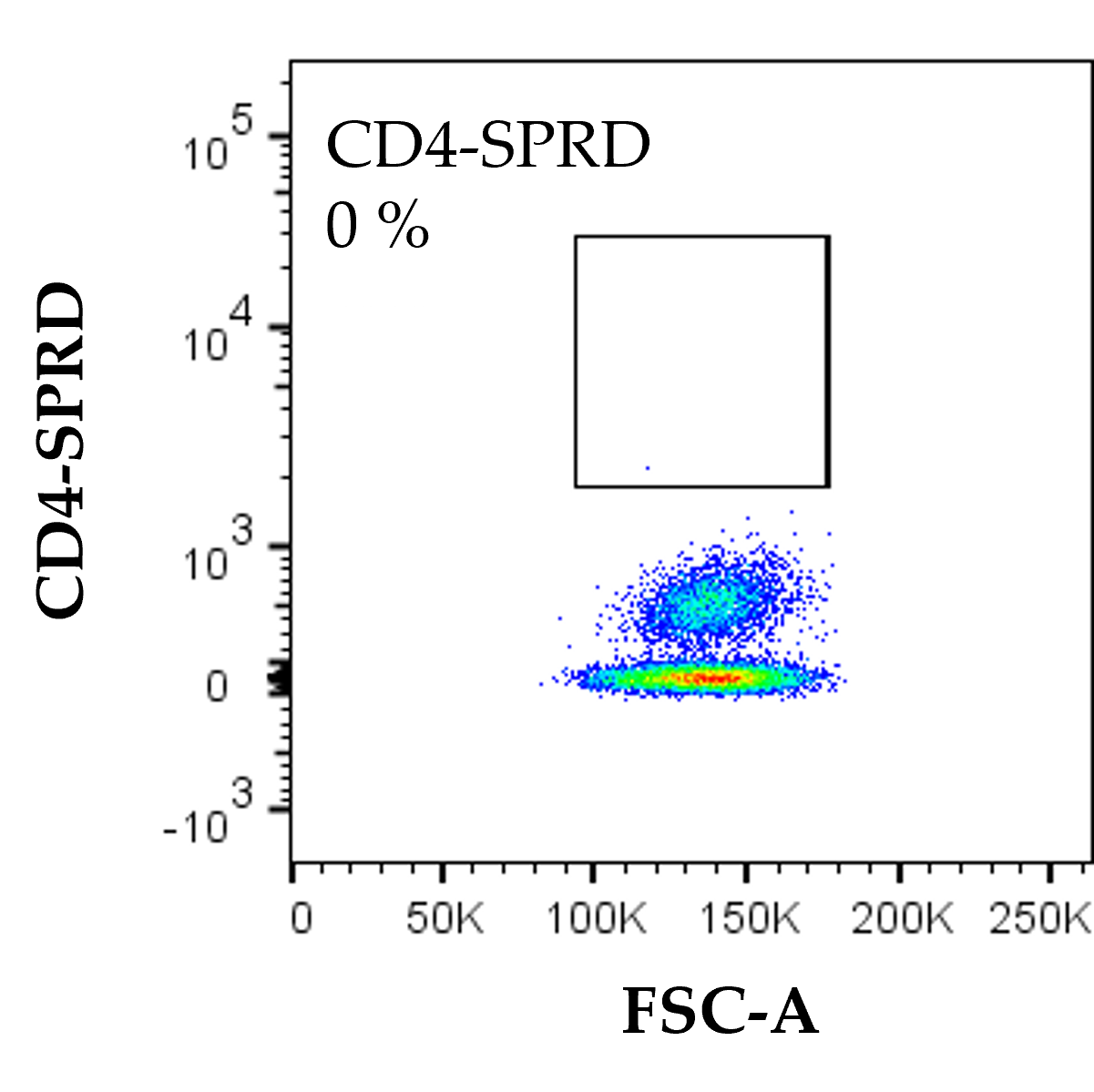

Supplement: Supplementary file 1 [file animals-11-03600-s001.zip › Figure S4f.png]

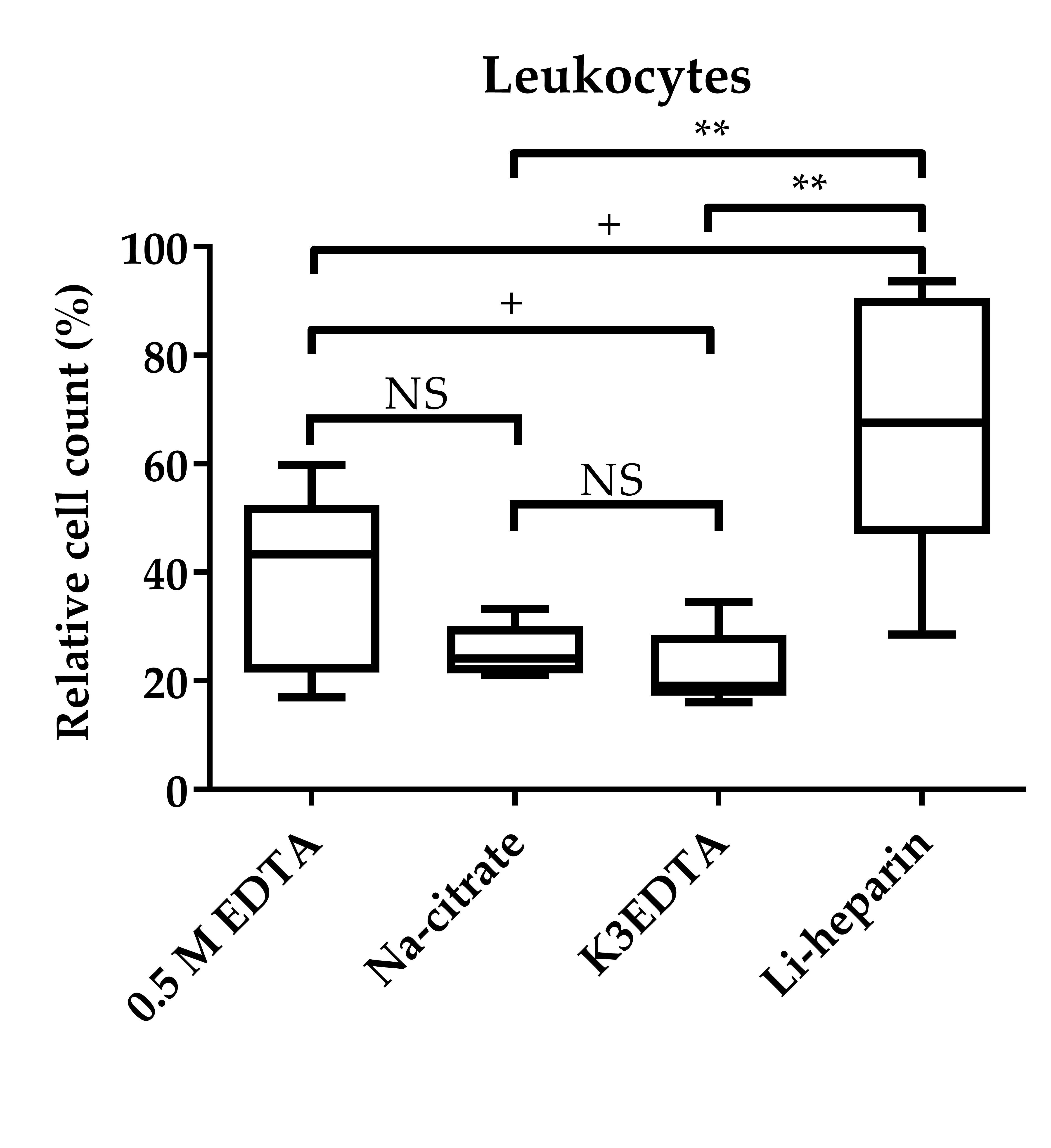

Supplement: Supplementary file 1 [file animals-11-03600-s001.zip › Figure S1a.jpg]

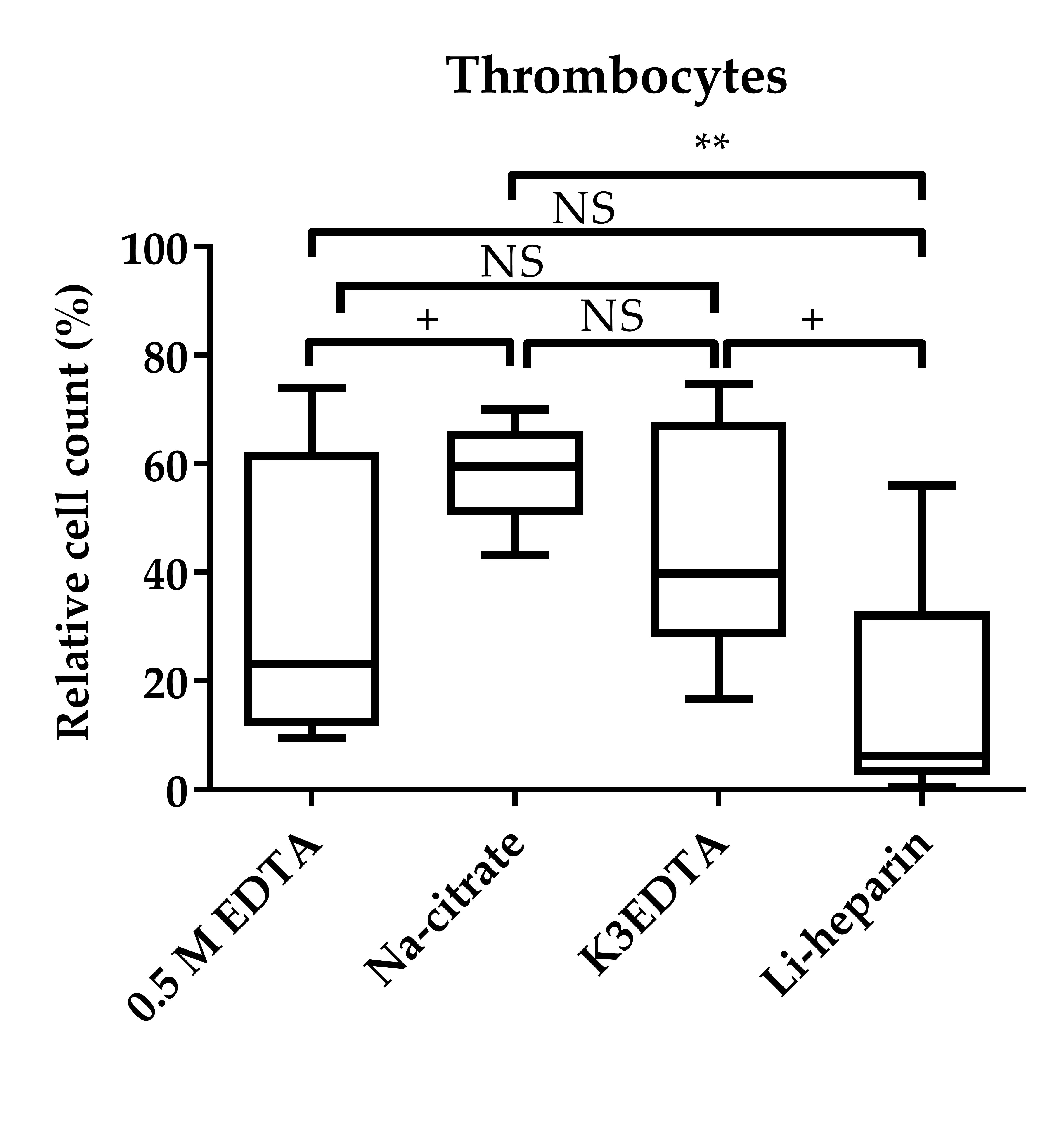

Supplement: Supplementary file 1 [file animals-11-03600-s001.zip › Figure S1b.jpg]

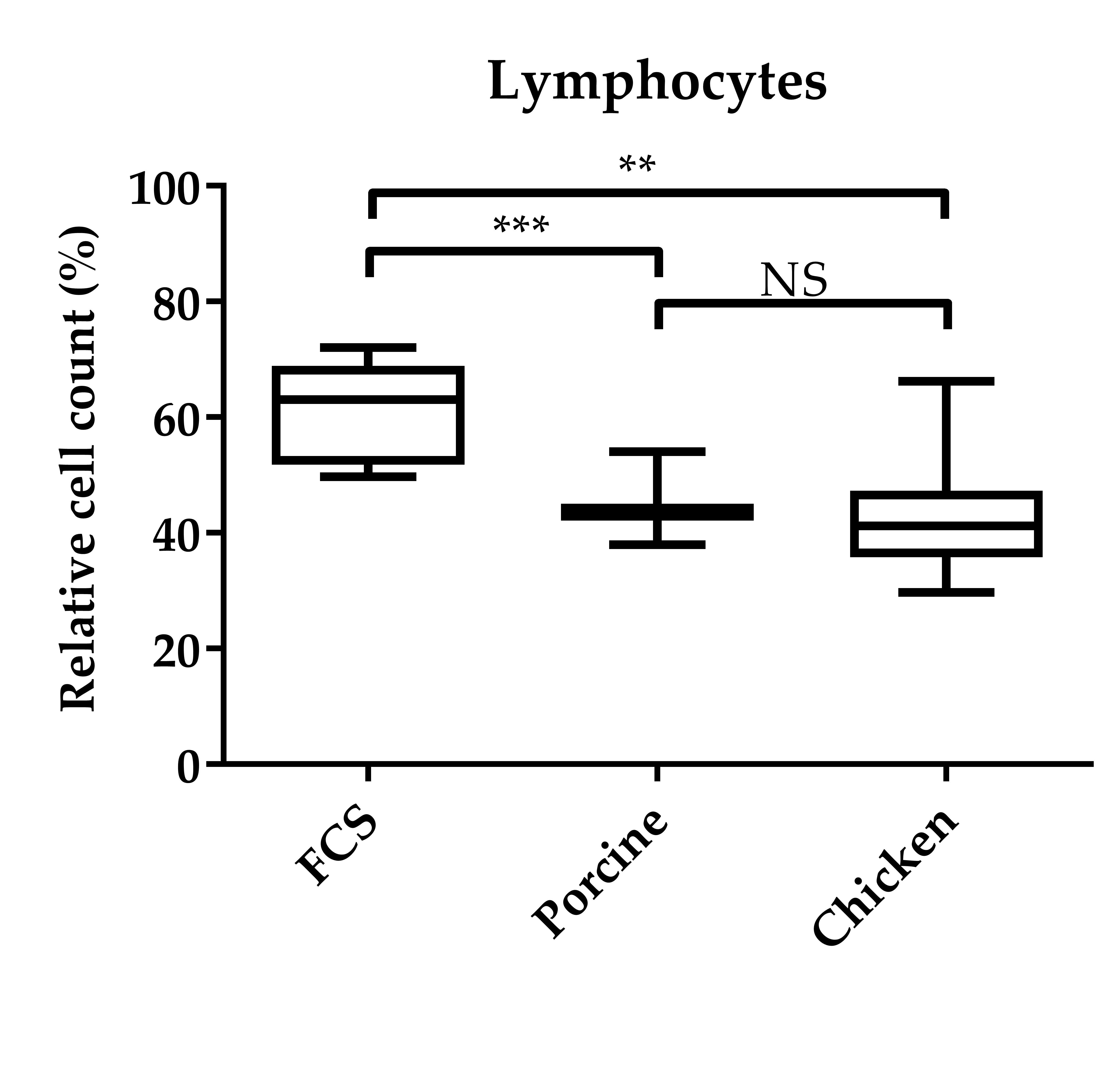

Supplement: Supplementary file 1 [file animals-11-03600-s001.zip › Figure S2a.jpg]

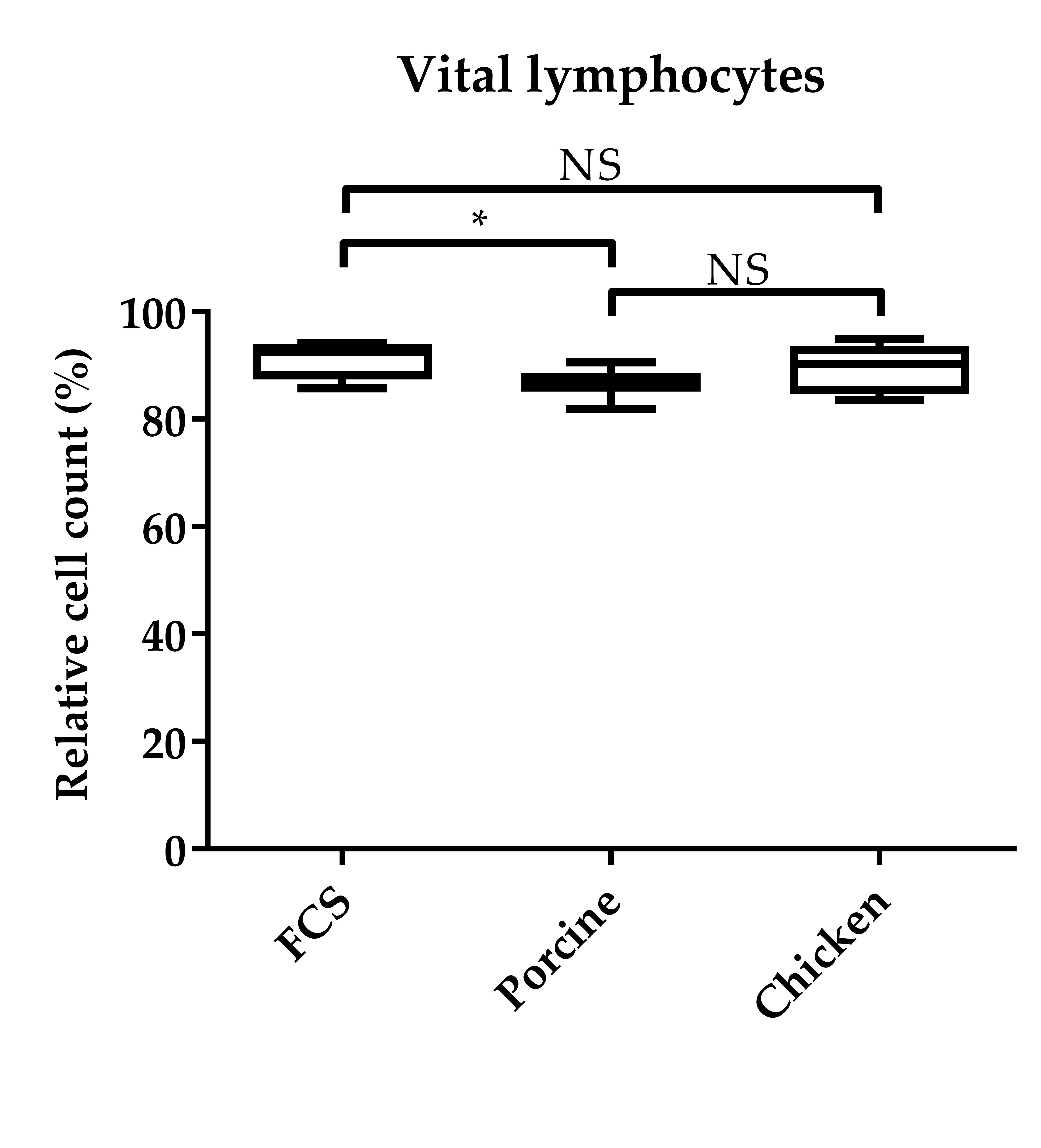

Supplement: Supplementary file 1 [file animals-11-03600-s001.zip › Figure S2b.jpg]

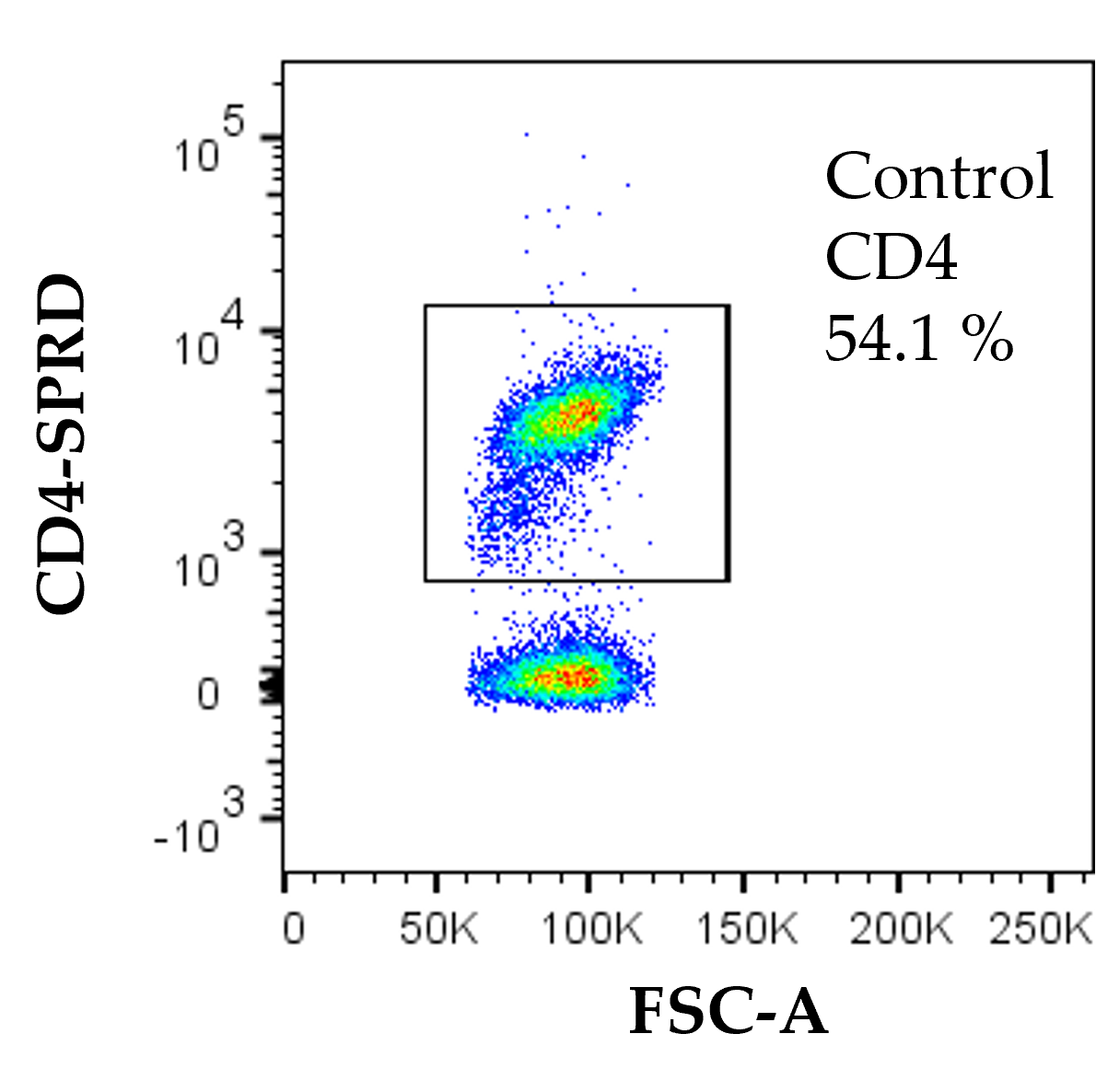

Supplement: Supplementary file 1 [file animals-11-03600-s001.zip › Figure S3a.png]

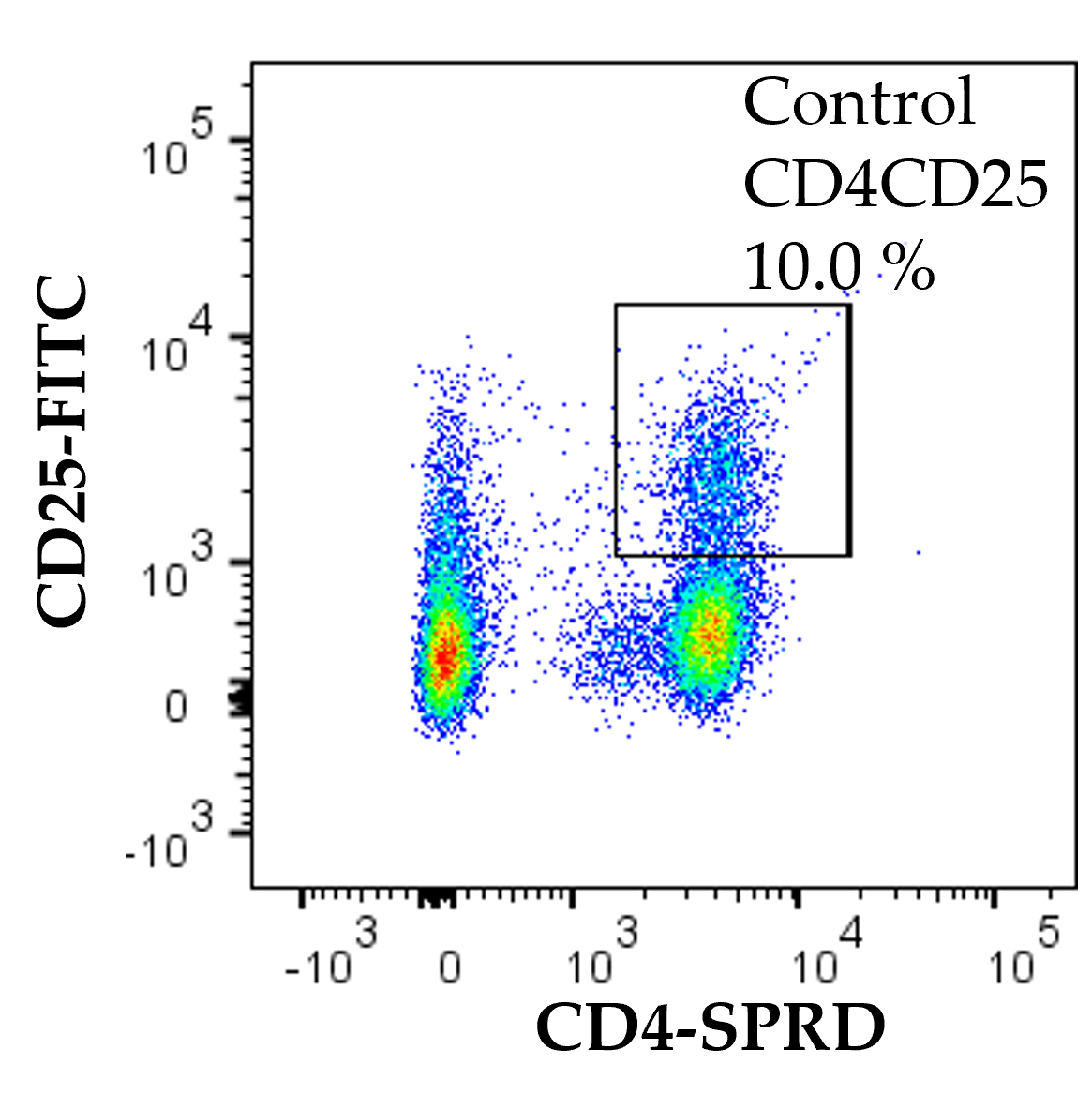

Supplement: Supplementary file 1 [file animals-11-03600-s001.zip › Figure S3b.png]

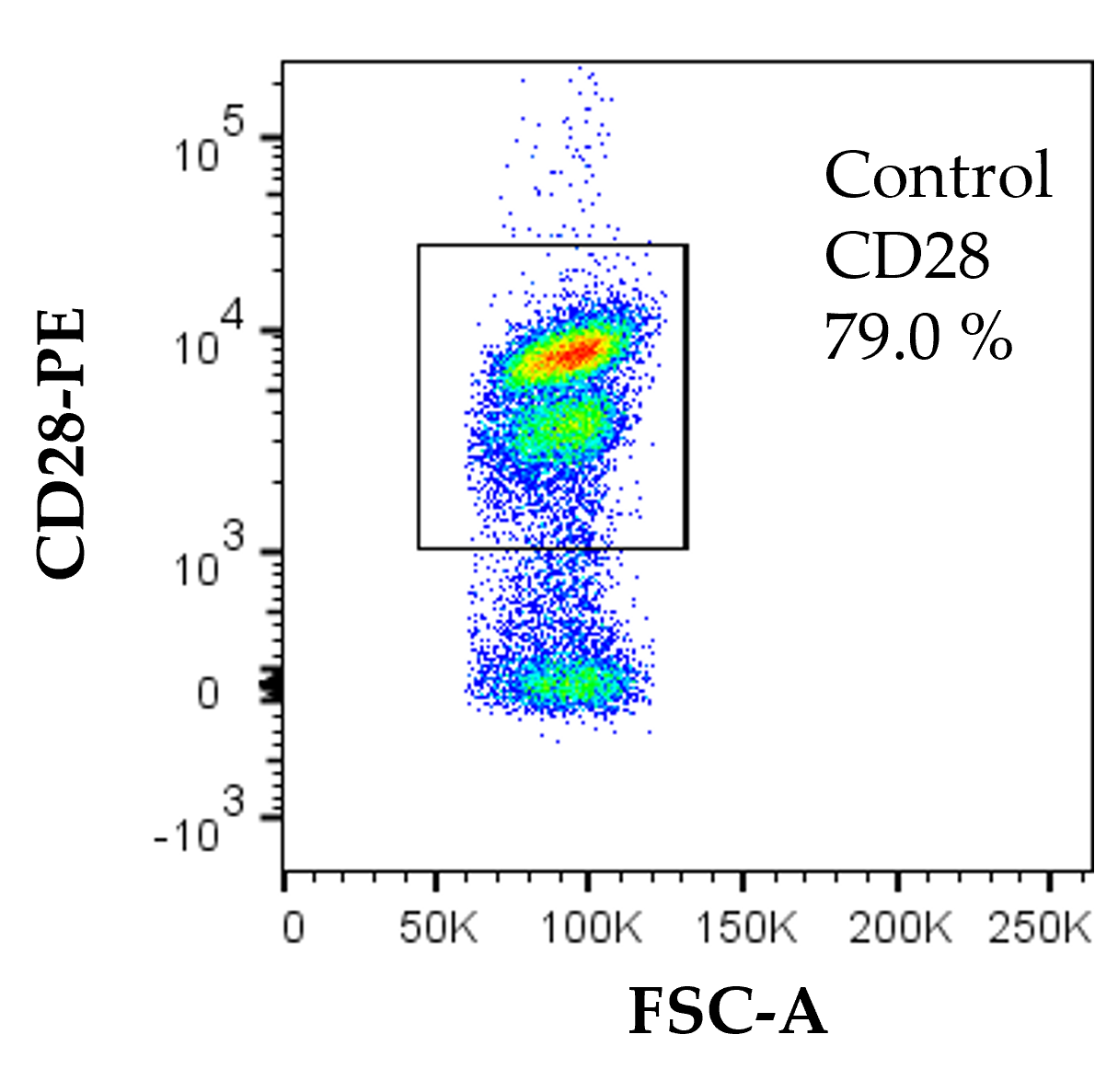

Supplement: Supplementary file 1 [file animals-11-03600-s001.zip › Figure S3c.png]

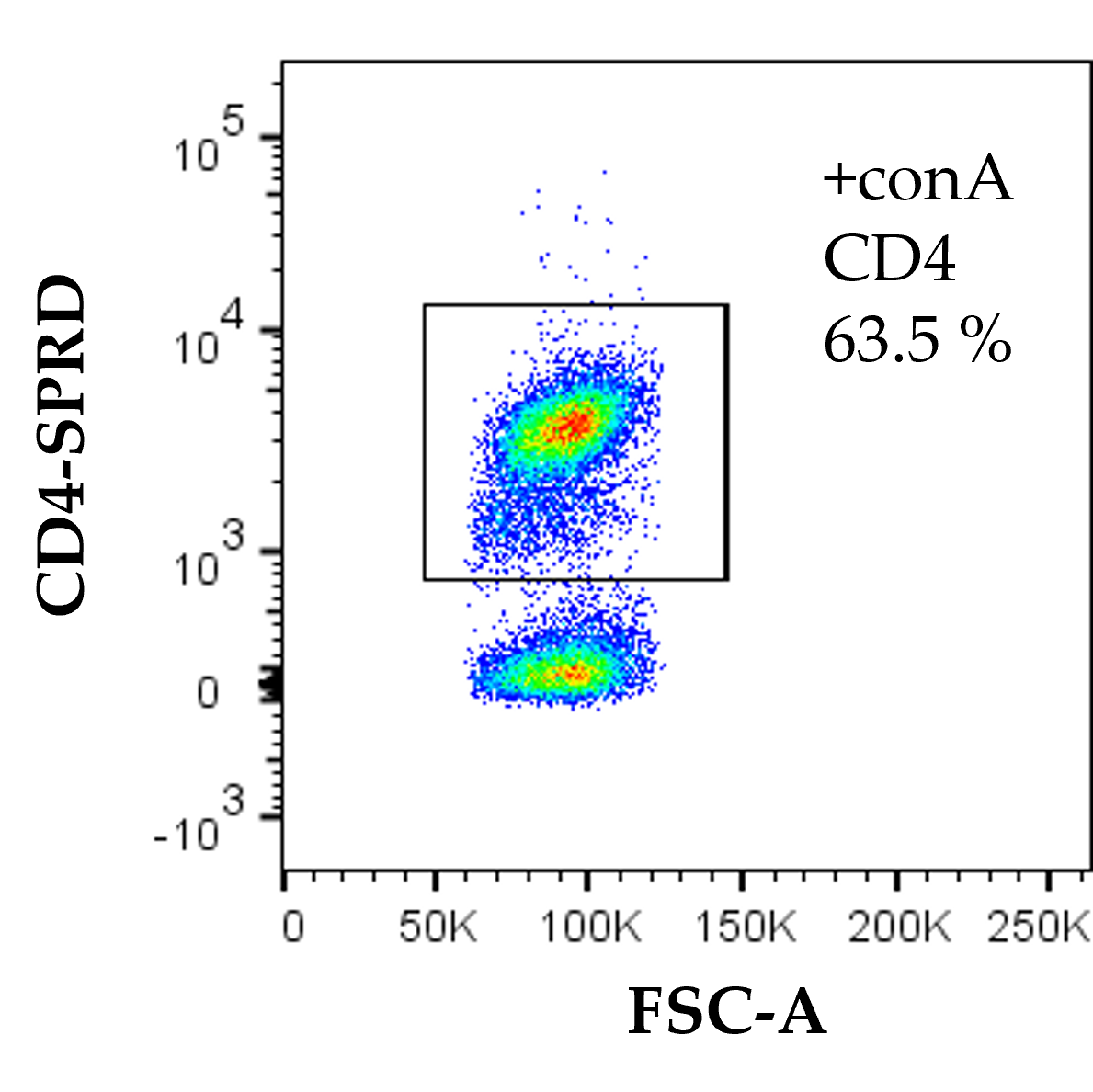

Supplement: Supplementary file 1 [file animals-11-03600-s001.zip › Figure S3d.png]

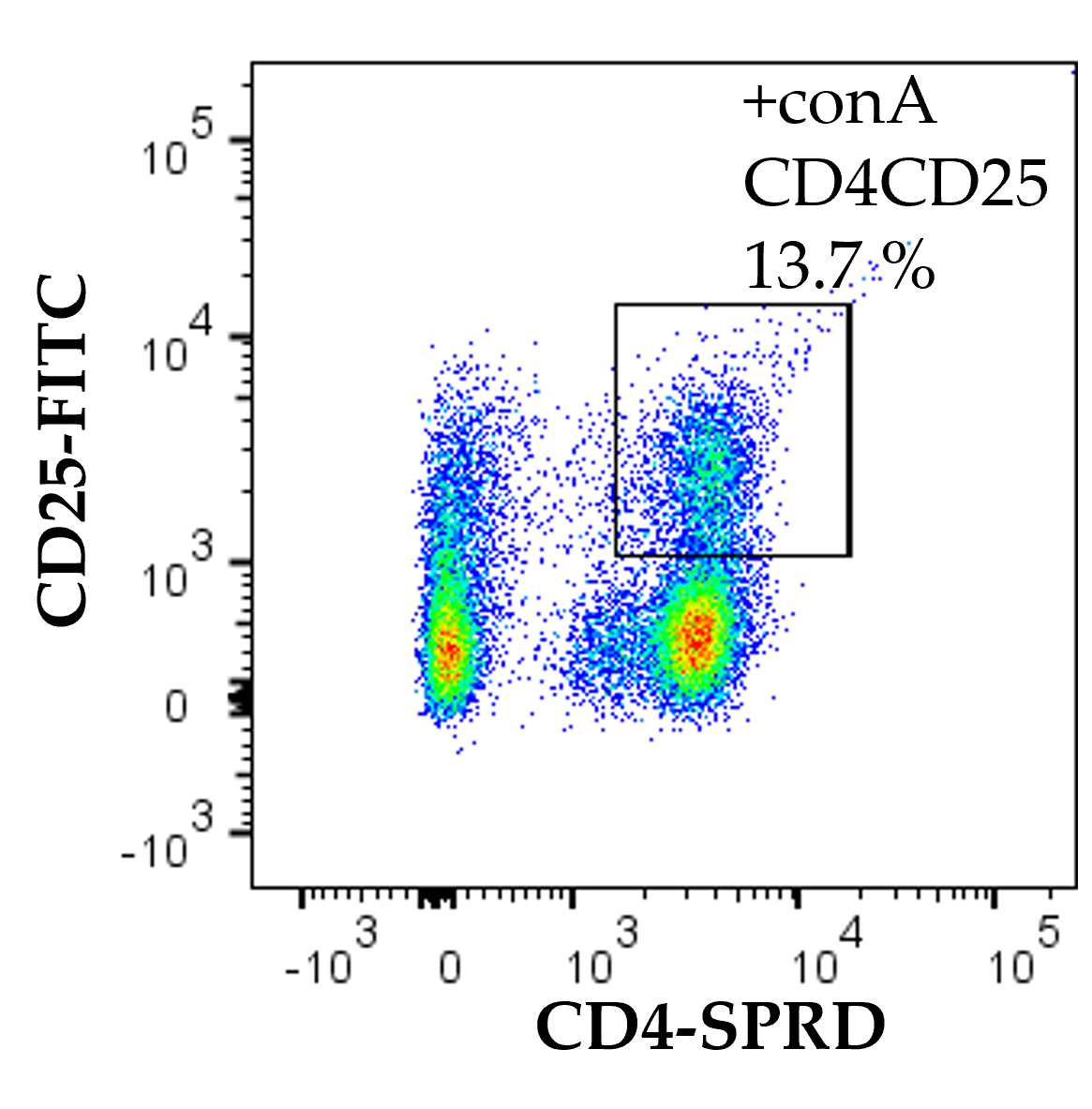

Supplement: Supplementary file 1 [file animals-11-03600-s001.zip › Figure S3e.png]

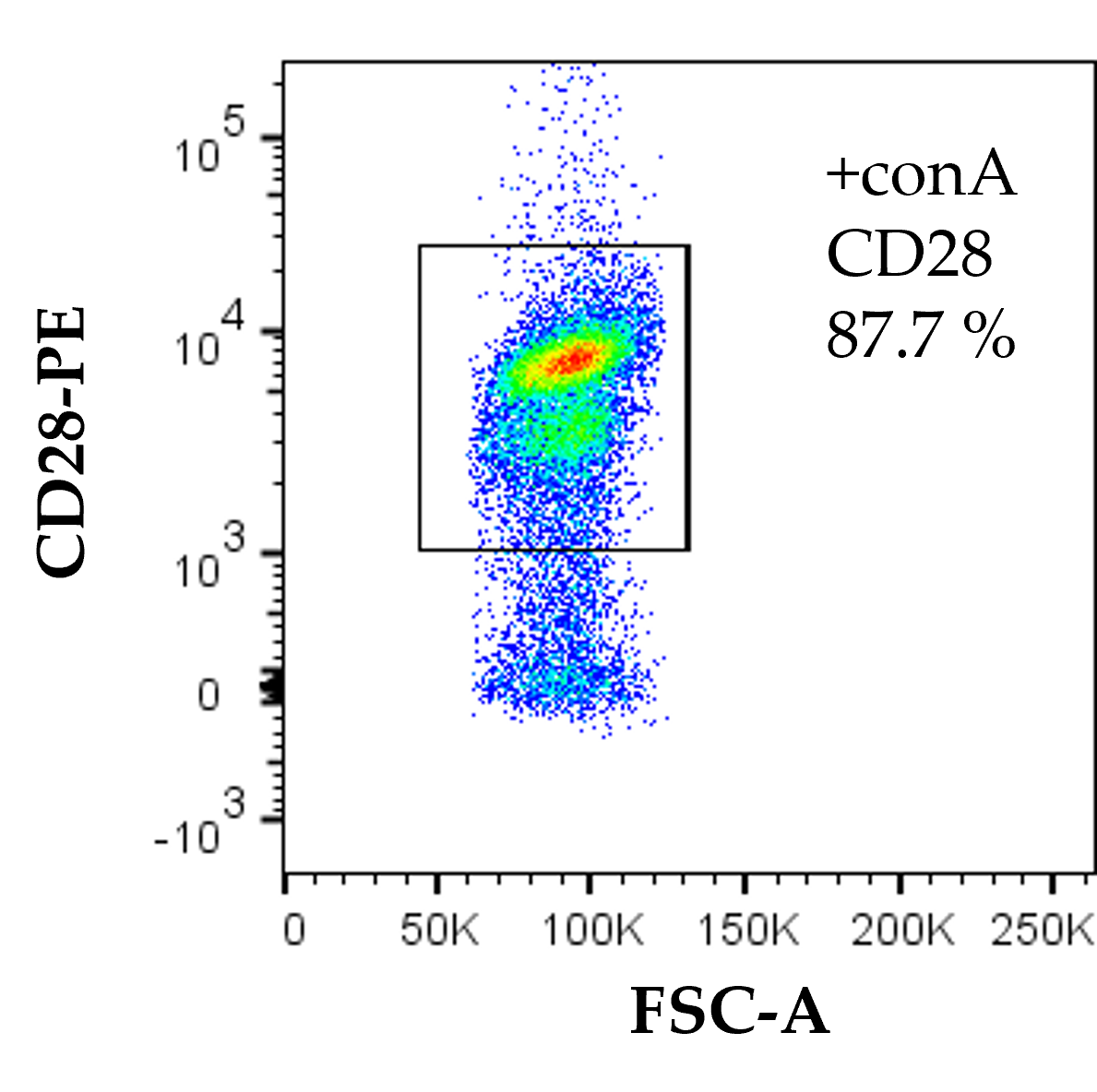

Supplement: Supplementary file 1 [file animals-11-03600-s001.zip › Figure S3f.png]

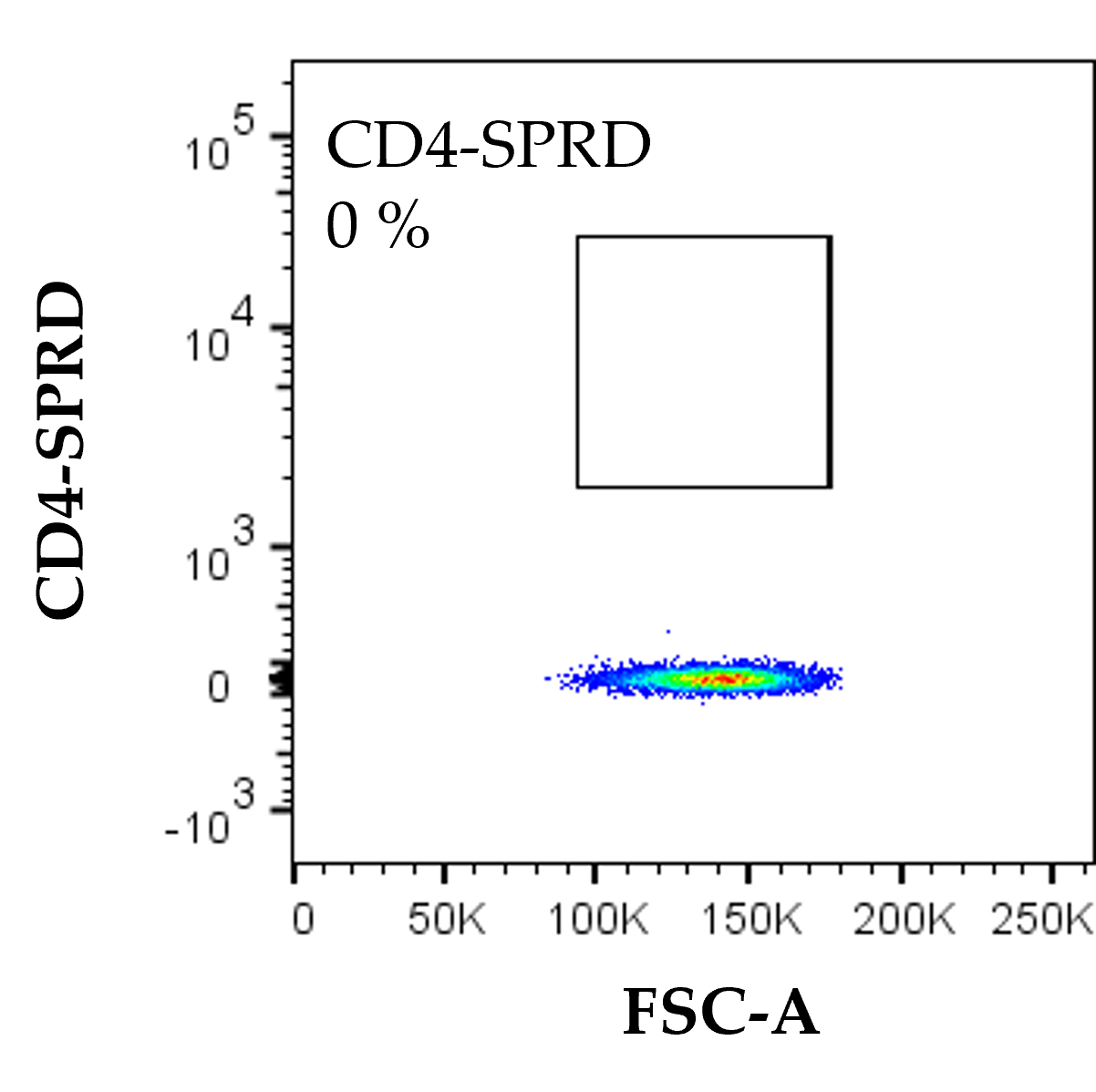

Supplement: Supplementary file 1 [file animals-11-03600-s001.zip › Figure S4a.png]

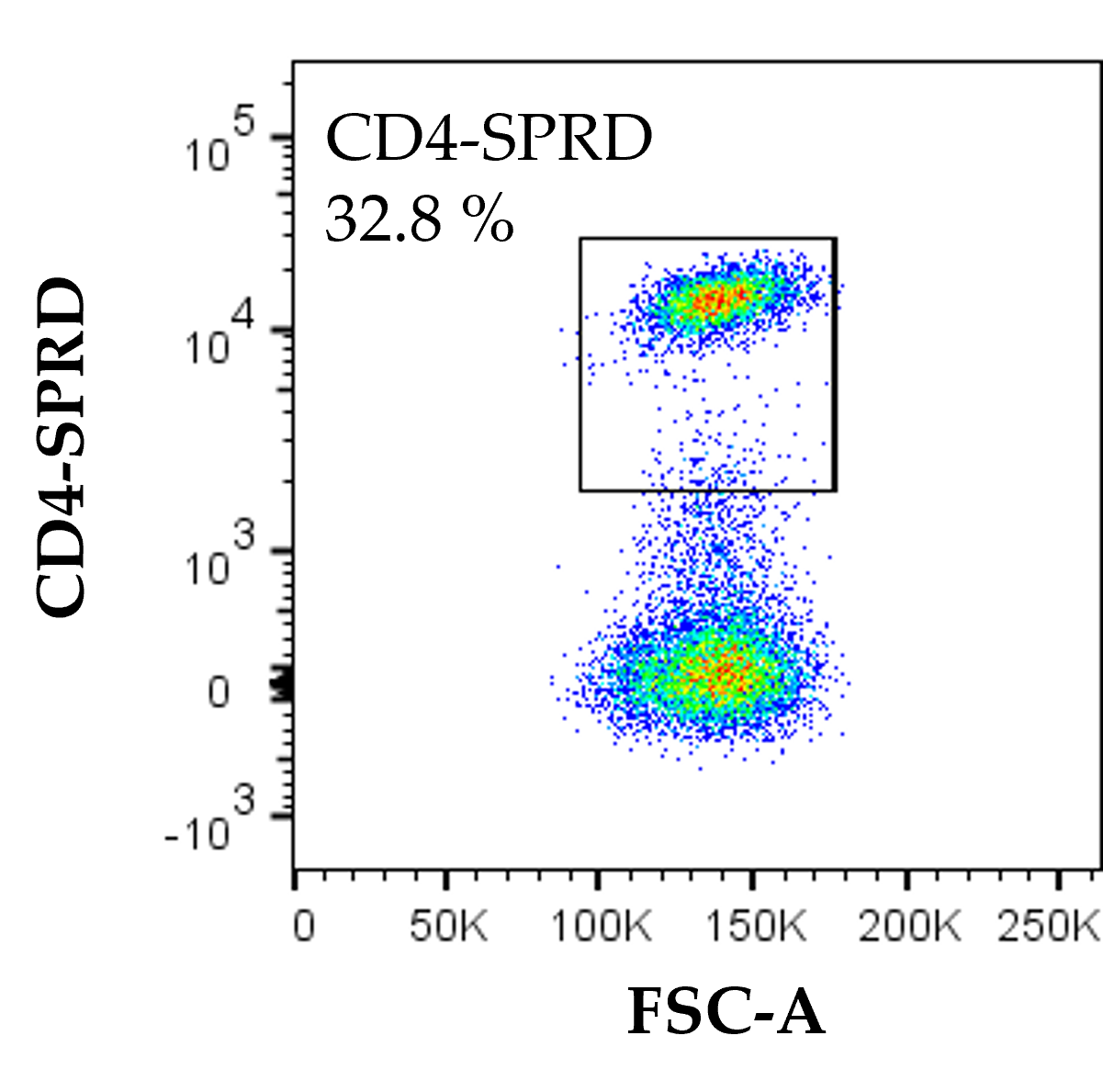

Supplement: Supplementary file 1 [file animals-11-03600-s001.zip › Figure S4b.png]

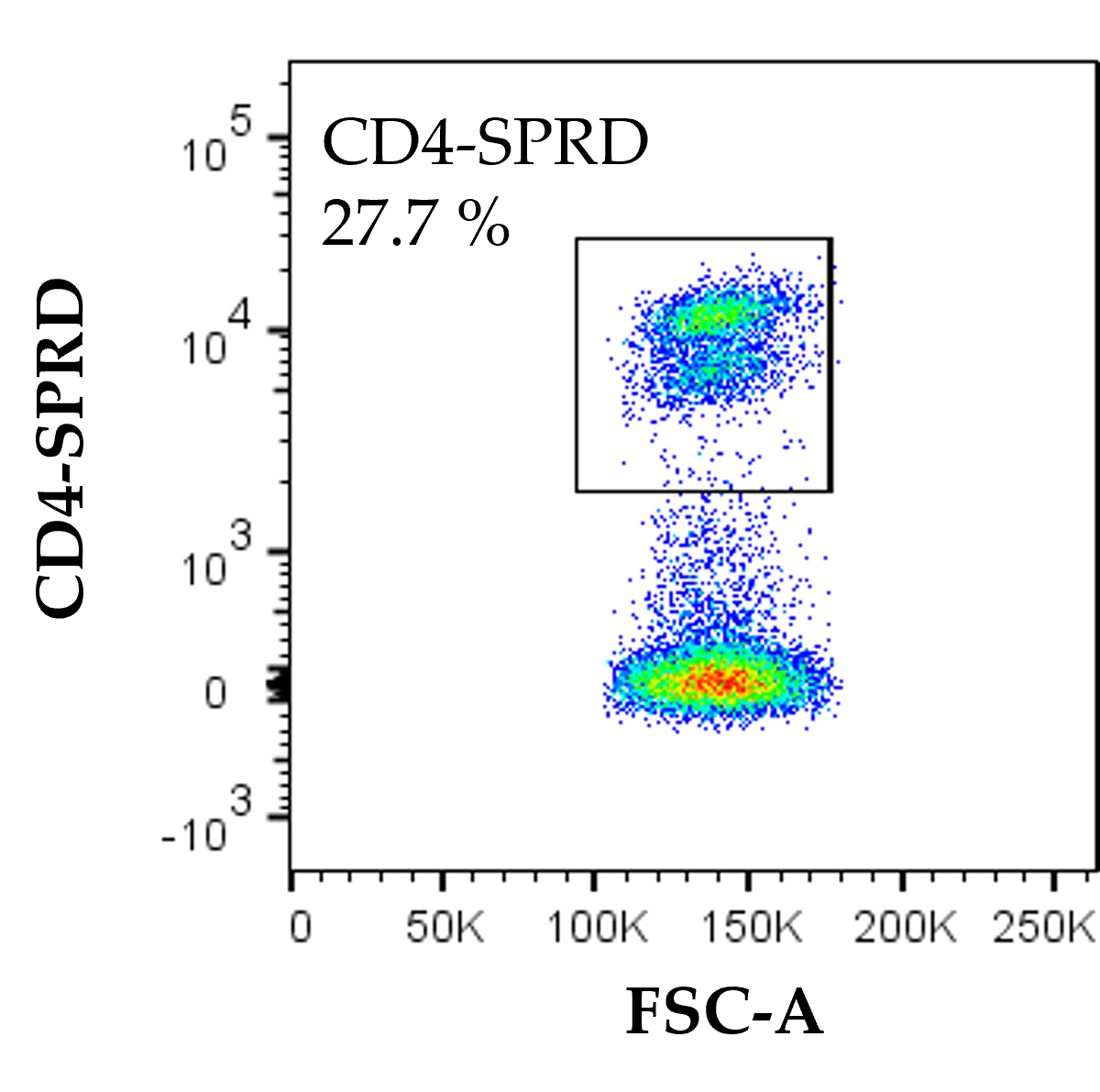

Supplement: Supplementary file 1 [file animals-11-03600-s001.zip › Figure S4c.png]

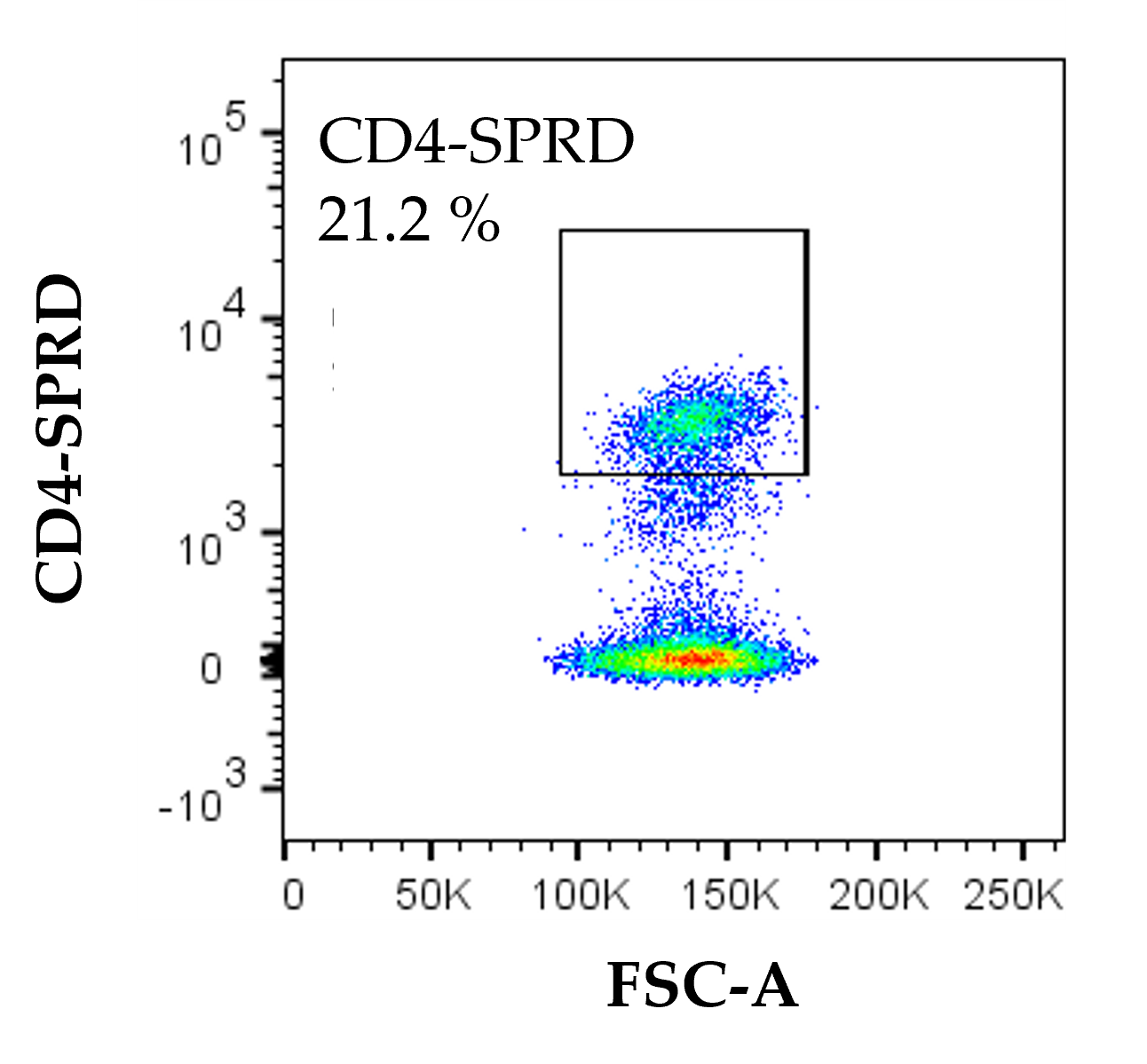

Supplement: Supplementary file 1 [file animals-11-03600-s001.zip › Figure S4d.png]

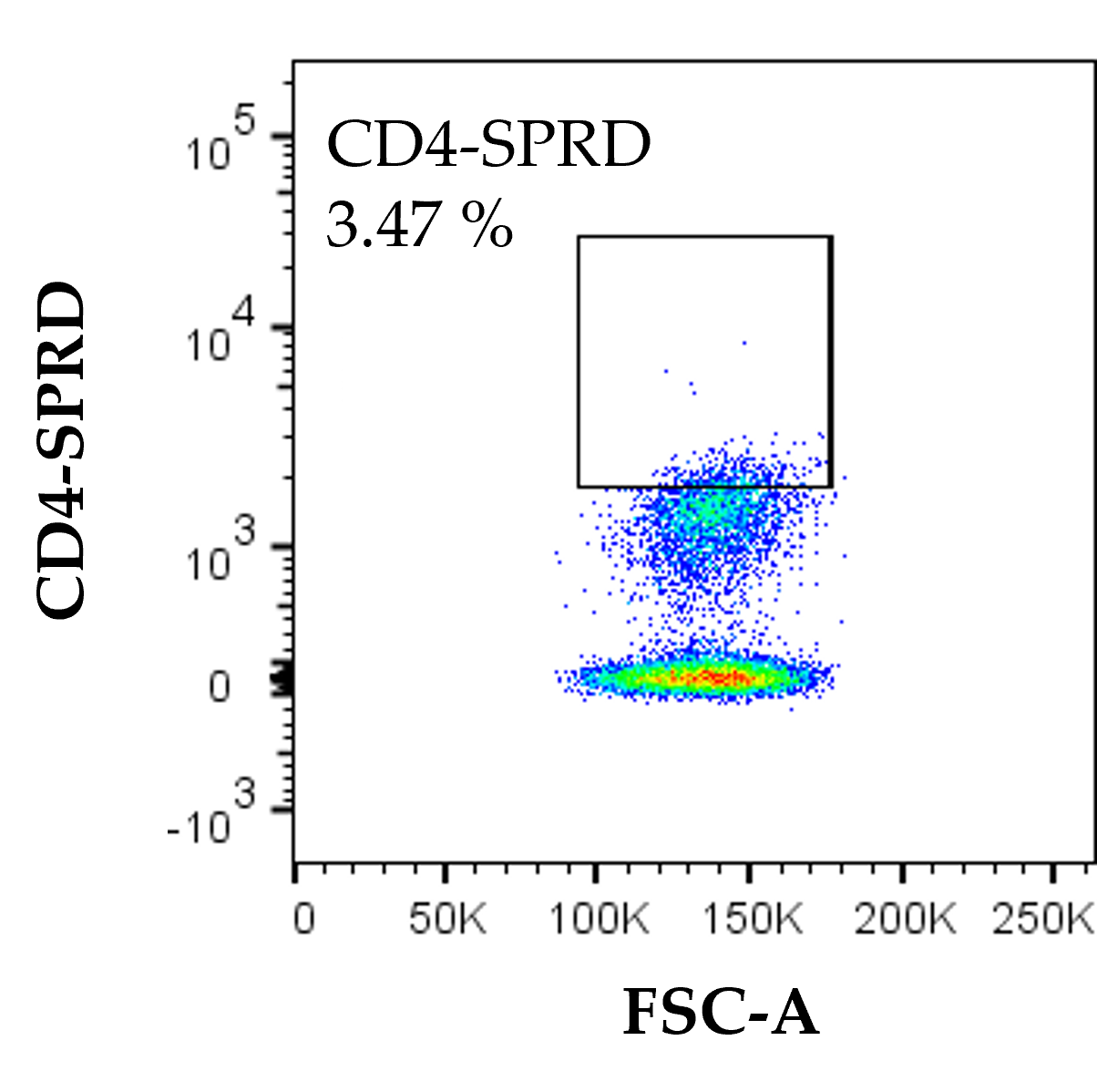

Supplement: Supplementary file 1 [file animals-11-03600-s001.zip › Figure S4e.png]
